# An empirical evaluation of electronic annotation tools for Twitter data

**DOI:** 10.5808/GI.2020.18.2.e24

**Published:** 2020-06-17

**Authors:** Davy Weissenbacher, Karen O'Connor, Aiko T. Hiraki, Jin-Dong Kim, Graciela Gonzalez-Hernandez

**Affiliations:** 1 Department of Biostatistics, Epidemiology and Informatics, Perelman School of Medicine, University of Pennsylvania, Philadelphia, PA 19104, USA; 2 Database Center for Life Science, Research Organization of Information and Systems, Kashiwa, Chiba 277-0871, Japan

**Keywords:** annotation tool, natural language processing, social media mining

## Abstract

Despite a growing number of natural language processing shared-tasks dedicated to the use of Twitter data, there is currently no ad-hoc annotation tool for the purpose. During the 6th edition of Biomedical Linked Annotation Hackathon (BLAH), after a short review of 19 generic annotation tools, we adapted GATE and TextAE for annotating Twitter timelines. Although none of the tools reviewed allow the annotation of all information inherent of Twitter timelines, a few may be suitable provided the willingness by annotators to compromise on some functionality.

## Introduction

Twitter is one of the leading social media platforms with more than 126 million daily users [1]. Twitter is now regarded by the natural language processing (NLP) community as a valuable source of information and has been the focus of a significant amount of research this last decade. An increasing number of shared-tasks have been organized utilizing data from this platform. Amongst the shared tasks for Twitter data, named entity recognition is well-represented, including the Named Entity Recognition and Linking Challenge series [2] which ran from 2013 to 2016, or the Workshop on Noisy User-generated Text series [3] which organized shared tasks from 2015 to 2017. Aside from named entity recognition, the community has extended its use of Twitter to broader tasks, such as the SemEval tracks on sentiment, opinion and abusive language classification starting in 2013 [4], or for health research with the Social Media Mining for Health (#SMM4H) running since 2016 [5]. Since more than half of tweets are not written in English, shared tasks are also utilizing corpora in various languages: the conference sur l'Apprentissage Automatique in 2017 in French [6], the Forum for Information Retrieval in 2016 in Indian [7], the Named Entity rEcognition and Linking in 2016 in Italian, a track in Arabic during SemEval 2017 and #SMM4H'20 with a task in French and Russian.

As the foundation for most shared tasks in NLP, and more generally most studies in NLP, the importance of the corpus cannot be overstated. A standardized corpus is essential for the evaluation of the competing systems. The correctness and consistency of the annotations are vital to ensure accurate results and predictions on how the systems will perform on unseen data. Moreover, with the generalization of statistical methods in NLP, annotations are also important for training the systems. Only well-defined, high-quality annotations can ensure that a machine learning-based system will be able to model discriminating patterns and perform correctly on a given task.

Despite the strong interest in Twitter data and the importance of creating high quality annotated corpora, few annotation tools have been developed specifically to handle these data. The only tool that we are aware of is described in Cresci et al.’s study [8] but it is not available to the community. Annotators are therefore forced to annotate Twitter data using third-party tools such as text editors/spreadsheets or adapting generic annotation tools such as GATE [9] or brat [10]. However, Twitter data have their specificities that generic tools do not account for, e.g. tweets are, most often, unrelated and posted over time by a user, making it difficult to annotate all pieces of relevant information needed across different tweets in a user’s timeline.

The three days of the Biomedical Linked Annotation Hackathon, BLAH6, was an opportunity for researchers to review existing annotation tools and evaluate their suitability for Twitter data. The four researchers involved in our project were given a real case corpus and they adapted two annotation tools, GATE and TextAE [11], to perform a predefined annotation task. We report in this study their evaluation according to predefined requirements and we discuss the functionalities that are still missing.

## Annotating Twitter Data

When registering for a Twitter account, a user is invited to fill a short description and choose other users to follow. The new user is assigned a unique user ID and each tweet posted by the user is identified by a unique tweet ID. In addition, each tweet is described by metadata such as the posting time or the predicted language of the tweet. The collection of all tweets posted by a given user is called the home timeline.

The four researchers participating in our project during the hackathon were provided with 25 timelines of women that had publicly announced their pregnancy on Twitter. These timelines correspond to a total of 74,016 tweets in English, with an average of 3,000 tweets per timeline. We defined 31 annotation types relevant to these pregnancies and manually pre-annotated the 25 timelines for the event.

With no annotation tool designed for Twitter timelines, we had to adapt an existing tool for this type of data. Before the hackathon, we listed a set of requirements a tool should fulfill to be usable with Twitter timelines and we asked our four participants to evaluate two annotation tools according to those specifications. The specifications are detailed in (Table 1).

## Adapting Existing Annotation Tools for Twitter

Prior to the publication of an extensive review of 78 annotation tools by Neve and Seva [12], we started a review of annotation tools for Twitter data. The inclusion criteria for our review were the availability and the ease of installation of the tools, or otherwise, a demonstration of the tool online. A tool was not easily installed when dependencies were missing, errors occurred, or external software, such as databases, needed. Among the 19 annotation tools we tested, few met the requirements we needed to perform timeline annotations. We had used the brat annotation tool for a previous project involving the annotation of PubMed Central articles; however, we found several problems with it when trying to use it for timeline annotations. Mainly, brat’s user interface was not adapted to annotate adjacent tweets. We reviewed a commercial application, LightTag [13], and though it provided a clean interface and supported many of our requirements, it crashed excessively during use. It also did not allow for subcategories of entity tags and, the tool not being open source, prevented us from modifying it to fit our needs. Other tools tested did not allow for the subcategorization of entity tags, including WebAnno [14], Yedda [15], and Slate [16]. These tools also did not provide support for the normalization of entities extracted. Supplementary Table 1 and 2 summarizes our review of the 19 tools. Our review found three possible annotation tools for our project eHost [17], GATE and TextAE, as they met most of our requirements. We chose the GATE, and TextAE annotation tools for the hackathon because they were actively supported and updated regularly.

## Tuning Gate for Twitter Data Annotation

GATE is an open-source toolkit developed for text annotation and automatic text processing. We used the stand-alone version of GATE to annotate Twitter timelines for prior projects [18]. Although a web-based version of GATE is available, GATE teamware [19], we compared TextAE with the stand-alone version of GATE, as we were already familiar with the tool and it was easier to install during the hackathon than the web-based version.

During the hackathon, we imported our 25 timelines and reviewed the tools with respect to our requirements. We imported a timeline as a unique document in GATE, one tweet per line. We inserted the tweet IDs and the posting dates before the text of the tweets to facilitate the annotation process, all items were separated by tabulations. Tweet IDs and dates were pre-annotated with their tags in the document. We named the file with the user ID. We could have added annotations at the timeline level (metadata), such as the gender or the place of residence of the user, by importing them as pre-annotation and inserting them at the beginning of the document in an empty span.

GATE fulfilled many of our specifications. GATE is actively supported, with its most recent release occurring on January 17, 2020. Written in Java and well documented, it is easy to setup. Pre-annotations and metadata can be imported provided that they are formatted in an XML file following a format specific to GATE. This XML format has been designed to support both nested and crossing annotations. GATE also supports subcategory annotations. Despite the large number of tweets in a timeline, GATE loads a timeline and its annotations in less than a second. It clearly marks completed annotations in the interface and offers the possibility to hide annotations when appropriate. GATE implements interfaces for active learning, but we did not use the service during the hackathon due to lack of time.

GATE appeared to be a valuable tool for annotating timelines but several drawbacks discourage us from using the tool for long term projects. There are some issues with the stability of the stand-alone version as GATE would crash occasionally. GATE also allows closing a file without saving the annotations and without warning the annotators. The internal XML format, specific to GATE, was difficult to work with and required the development of scripts to convert pre-annotated timelines in order to import them in GATE as well as to export the timeline annotated for further use in external applications. Whereas annotations at the tweet level were well supported, annotating timelines was only possible as pre-annotation and the built-in GATE User Interface would not allow annotators to edit these annotations. Due to the time constraint, we did not evaluate the diff tool plugin [21] to compute the inter-annotator agreement in GATE. The format of the output also made it difficult to manually perform these two tasks.

## Tuning PubAnnotation/TextAE for Twitter Data Annotation

TextAE is a web-based interface designed for corpus annotation. The interface is integrated with PubAnnotation [22], a public repository for literature annotation. For the hackathon, we chose the public version of PubAnnotation to create a private project, eliminating the need of a local installation and enabling the storage of our data in the cloud. We imported 5 timelines, representing the timelines in the same way as we did in GATE, one timeline per document, one tweet per paragraph, the document named with the user ID, and tweet IDs/posting dates inserted before the texts of the tweets.

The current versions of PubAnnotation/TextAE do not meet some of our requirements and would be too limited for our usage. However, the tools are still under development and, with the improvements scheduled, they could become standard for annotating Twitter data. An annotation project can be set up in PubAnnotation for multiple annotators as a collection, with a project created for each annotator. Annotation tags are created using a JSON configuration file. The TextAE interface allowed each tweet to be loaded as a paragraph. The annotation interface was not intuitive for all users. However, with documentation online, most annotators were readily able to access text and begin annotating within an hour. Some choices in the ergonomic design of the annotation interface were not optimal for our task and added time to the annotation process. The interface displays the annotated texts with the labels appearing on top of the text. The tool supports nested annotations. Although it is possible to add multiple annotations on the same span of text, this functionality was unstable in the version evaluated. Annotating the span in the wrong order resulted in the loss of the top-level annotation. The tool does not support crossing annotations since it uses HTML to display annotations and HTML does not allow crossing tags. TextAE could annotate a continuous set of tweets but this will require minor changes in the tool. TextAE does not currently support timeline annotations, but plans were made during the hackathon to extend the interface to add and edit this level of annotations. TextAE, combined with PubDictionaries, allows subcategories and normalization of annotations to standardized terms. Despite the size of our timelines (3,828 tweets, 418 KB, on average) and the dictionary used for testing the normalization (two million entries, 132 MB), both tools reacted within the time constraints imposed by our requirements. TextAE also provides an interface for the comparison of documents annotated by multiple annotators for disagreement resolution. TextAE was stable when annotating our timelines and, although the annotations must be manually saved, there is a warning presented to the annotator before closing. Annotations are saved in a separate file that can be exported as JSON or TSV files. Given the time limit of the hackathon, we did not test the import functionality in JSON format. An active learning API for the tool is in development and was not ready during the event.

## Conclusion

The need for annotation tools dedicated to Social Media data, such as Twitter, is becoming more apparent as the interest of the NLP community is growing for this data. Since, to the best of our knowledge, there is no annotation tool dedicated to Twitter available, we evaluated during the 6th edition of the Biomedical Linked Annotation Hackathon two generic annotation tools using 25 Twitter timelines as a way to test their functionalities. After defining a catalog of requirements for an annotation tool dedicated to Twitter, we reviewed 19 tools and selected GATE and TextAE/PubAnnotation for our evaluation. Our results show that, whereas neither of them allows the annotation of all information characterizing Twitter timelines, each may be adapted for this purpose, if annotators are willing to compromise on some functionalities.

## Figures and Tables

**Table 1.** Requirements for an annotation tool dedicated to Twitter data

Requirement	Description
Accessibility	*The annotation tool should be web-based to support for multiple annotators and to enable inter-annotator agreement calculation and disagreement resolution. *Web-based tools, such as GATE teamware or brat, make it easier to manage a team of annotators and compute the inter-annotator agreement.
Set up	*It should be easy to install, to set up the tags and the annotation schema as well as allowing changes to the schema. *Twitter data are used for various research projects, each project mining for different types of information requiring their own annotation schemas (e.g., normalizing adverse drug reaction (ADR), extracting reasons of drug non-persistence, etc.)
Efficiency	*It should load the tweets composing a timeline in less than 2 seconds and load an external dictionary for normalizing an annotation in less than 3 seconds. *A dictionary may be opened several times per tweet to normalize annotations, such as ADRs. A reading time longer than 3 seconds may significantly slow down the annotation of large corpora.
Stability	*It should not present recurrent bugs preventing or modifying the annotation process. The tool should be actively supported. *Active support would ensure the correction of such bugs.**
Auto-saving	*It should periodically save the annotated document and save automatically upon closing the document or, in the absence of automatic saving, warn the annotators to save before closing.* When annotating long documents such as timelines, annotators are likely to close a document without saving, losing their annotations.
Import	*It should allow the upload of pre-annotated labels and metadata (e.g., tweet IDs or date of post). The import formats should be standard like XML or JSON. *Non-standard formats, such as the XML format used in GATE, required developing conversion scripts to process new corpora.
**S**tand-off annotations	*It should store the annotations in a separate file, leaving the original document intact. *Stand-off annotations are preferred because**corpora may be used for different projects (e.g., timelines collected to study adverse pregnancy outcomes reused to study topics discussed during pregnancy)
Multi-level annotations	*It should allow for nested and crossing annotations.* Two annotations are nested if the span of one annotation is included in the span of the second annotation; they cross if they share a common span of text.
Annotation spans	*It should allow for annotating various levels of a timeline, the timeline itself, and the network of a Twitter user.* These levels are annotating spans of a tweet (e.g., the name of a drug), the tweet itself (e.g., the sentiment of the tweet), continuous set of tweets, i.e., an annotation spanning over multiple and adjacent tweets (e.g., all tweets posted by a user in May 2016).
Readability	*The interface should present a timeline to the annotator in a way that all annotations are easily distinguishable from each other and from the span annotated. Annotations should appear above the span annotated. The metadata should be included in the annotation file but not visible in the timeline during annotation. *Most research projects involve annotating multiple types of annotations, e.g., annotating a drug name and annotating if the drug was taken. Annotations are likely to overlap, cluttering up the document without a well-designed user interface.
Subcategories	*It should support for defined entity tags to have assignable subcategories*. For example, annotating alcohol intake, subcategories could be: intake, possible_intake, no_intake.
Normalization	*It should support the inclusion of a dictionary or ontology for normalizing the annotated entities to standardized terms.* For example, normalizing the annotated span ‘sleepy’ by linking it to the MedDRA preferred term ‘Somnolence’.
Active learning	*It should provide a default API to plug in an external classifier implementing an active learning algorithm to assist the annotation process.* The classifier could, for example, pre-annotate the sentiments of tweets. Using active learning, it can ask an annotator to correct the labels it assigned with less certainty and retrain its model after the labels are corrected. After some iterations, the classifier should annotate most of the tweets with the correct sentiments, saving manual annotation time compared to manually annotating all tweets [20].
Multi-annotator support	*It should calculate the inter-annotator agreement and provide an interface to help adjudication.*
Export	*It should support the export of the annotations in standard formats such as JSON, TSV, XML, etc.*

## Supplementary Materials

Supplementary Table 1. Requirement review of annotation tools for Twitter

Supplementary Table 2. Annotation tools reviewed
