# Supplementary material for: An empirical evaluation of electronic annotation tools for Twitter data
Source: Genomics Inform. 2020 Jun 17;18(2):e24. doi: 10.5808/GI.2020.18.2.e24 (PMC7362942; doi:10.5808/GI.2020.18.2.e24)
Supplement: Supplementary Table 1. — Requirement review of annotation tools for Twitter [file gi-2020-18-2-e24-suppl1.pdf]

Supplementary Table 1. Requirement review of annotation tools for Twitter

Annotation Tool	Accessibility	Active learning	Export	Import	Multi-annotator support	Multi-level annotation	Normalization	Set-up	Subcategories	Stability
Anafora[a]	Met	Not met	NR	NR	Not met	Not met	Not met	NR	Not met	NR
Brat	Met	Not met	Partially met	Partially met	Met	Met	Met	Met	Met	Met
Djangology[b]	Met	Not met	NR	NR	Met	Met	Not met	NR	Not met	NR
Doccano[a]	Met	Not met	NR	NR	Met	Not met	Not met	NR	Not met	NR
eHost	Not met	Not met	Partially met	Partially met	Met	Met	Met	Met	Met	Met
GATE	Met	Not met	Partially met	Partially met	Partially met	Met	Met	Met	Met	Not met
Inception[a]	Met	Met	Met	Met	Met	Met	Met	Met	Not met	NR
Lighttag	Met	Met	Partially met	Partially met	Met	Met	Not met	Not met	Not met	Not met
MAE	Not met	Not met	Partially met	Partially met	Not met	Not met	Not met	Met	Not met	Partially met
Slate	Not met	Not met	Partially met	Partially met	Not met	Met	Not met	Met	Not met	Partially met
Tagtog	Met	Met	Met	Not met	Met	Not met	Met	NR	Not met	NR
WebAnno[a]	Met	Not met	Met	Met	Met	Met	Met	Not met	Not met	NR
Yedda	Not met	Not met	Met	Not met	Met	Not met	Not met	Partially met	Not met	Met

□ = Requirement not met ▒ = Requirement partially met ■ = Requirement Met NR = Not Reviewed

[a]Our review was done based on the software demonstration.

[b]No demonstration available, our review was done based on the documentation.

We were unable to run 3 of the 19 tools due to installation errors or dependency issues, Argo, Callisto, and Knowtator. Three others, Pubtator, BioQRator, and ezTag, required a specific input file format, such as BioC, and therefore were not suited to annotate tweets. In Supplementary Table 1, we summarize our review of the features of the remaining 13 annotation tools for the most important requirements in our catalog. Note, as we were just reviewing the features of the tools, we did not complete full installations for the tools with external dependencies such as server and/or database installations to run. For those tools, we examined the online demonstrations if available during our assessment.
