# Supplementary material for: An empirical evaluation of electronic annotation tools for Twitter data
Source: Genomics Inform. 2020 Jun 17;18(2):e24. doi: 10.5808/GI.2020.18.2.e24 (PMC7362942; doi:10.5808/GI.2020.18.2.e24)
Supplement: Supplementary Table 2. — Annotation tools reviewed [file gi-2020-18-2-e24-suppl2.pdf]

Supplementary Table 2. Annotation tools reviewed

Annotation tool	URL
Anafora	https://github.com/weitechen/anafora
Argo	http://argo.nactem.ac.uk
BioQRator	http://www.bioqrator.org
Brat	https://brat.nlplab.org
Callisto	https://mitre.github.io/callisto
Djangology	https://sourceforge.net/projects/djangology
Doccano	http://doccano.herokuapp.com
eHost	https://github.com/chrisleng/ehost
ezTag	https://eztag.bioqrator.org
GATE	https://gate.ac.uk/teamware
Inception	https://inception-project.github.io
Knowtator	https://protegewiki.stanford.edu/wiki/Knowtator
Lighttag	https://www.lighttag.io
MAE	http://keighrim.github.io/mae-annotation
Pubtator	https://www.ncbi.nlm.nih.gov/research/pubtator
Slate	https://github.com/jkkummerfeld/slate
Tagtog	https://www.tagtog.net
WebAnno	https://webanno.github.io/webanno
Yedda	https://github.com/jiesutd/YEDDA
